# Beyond “What Should I Eat to Get Better?”: Medical Literacy, Dietary Narratives, and Supportive Nutrition in Hematologic Diseases

**DOI:** 10.31662/jmaj.2026-0074

**Published:** 2026-05-08

**Authors:** Akiyoshi Takami, Ichiro Hanamura

**Affiliations:** 1 Division of Hematology, Department of Internal Medicine, Aichi Medical University School of Medicine, Nagakute, Japan

**Keywords:** hematologic diseases, health literacy, misinformation, supportive care

## Abstract

Patients newly diagnosed with hematologic diseases frequently ask, “What should I eat to get better?” This question reflects both a deeply rooted societal narrative linking lifestyle to recovery and a desire for personal agency in moments of vulnerability. Nutrition remains an essential component of supportive care. However, for most malignant and clonal hematologic disorders, there is limited clinical evidence that specific diets or commercially marketed supplements alter disease biology or replace standard therapy. In the digital era, dietary narratives are amplified by social media, online advertising, and commercial interests, increasing the risk of misinformation and exploitation. We argue that clinicians and professional societies should strengthen public medical literacy, clearly distinguish supportive nutrition from disease-modifying therapy, and proactively protect patients from misleading health claims.

## A Recurrent Question at the Time of Diagnosis

For more than three decades, as clinical hematologists and academic physicians, we have observed that patients newly diagnosed with hematologic diseases, whether acute leukemia, marrow failure syndromes, or other hematologic malignancies, almost invariably ask: “What should I eat to get better?”

This question is rarely casual. It is typically voiced in visible distress, immediately after disclosure of a potentially life-threatening diagnosis. Its near universality suggests that it reflects a broader societal narrative linking diet, lifestyle, and recovery. Patients who ask this question are heterogeneous. Some seek reassurance and a sense of agency; others wish to complement medical therapy; and some are already influenced by media or alternative health information. Recognizing these differing motivations is essential for appropriate clinical communication.

## Medical Literacy, Health Literacy, and the Clinical Task

Health literacy is the established framework for describing a person’s ability to access, understand, appraise, and use health information. In this article, we use the term medical literacy in a narrower and more practical sense. We refer to the capacity―supported and often scaffolded by clinicians―to place health information into medically meaningful categories, especially the distinction between supportive care and disease-modifying therapy. This usage overlaps with critical health literacy, particularly in digital environments, but it emphasizes a concrete clinical task at the time of diagnosis: helping patients judge whether a dietary claim is supportive, speculative, misleading, or potentially harmful.

Because dietary advice is culturally familiar and emotionally resonant, it can easily be perceived as therapeutically equivalent to antineoplastic or other disease-directed treatment. Clarifying that it is not equivalent is therefore not a semantic exercise; it is a patient-safety function.

## Lifestyle Narratives and Their Extension to Hematologic Disease

Many patients with cancer turn to complementary or alternative medicine, often in parallel with conventional therapy and occasionally in its place. However, observational evidence suggests that replacing standard treatment with alternative approaches may be associated with inferior survival outcomes in certain settings ^(1)^.

For most malignant and clonal hematologic disorders, credible clinical evidence supporting specific foods or supplements as disease-modifying therapy is lacking. This point is especially important for non-specialist readers: our concern here primarily relates to malignant and clonal disorders, not to clearly defined nutritional deficiency states. Certain hematologic abnormalities, such as vitamin B_12_ or copper deficiency, respond predictably to nutritional correction. Those biologically defined conditions differ fundamentally from clonal malignancies and therefore, should not be conflated with them.

## Scientific Evidence and Its Limits

Structured nutritional support remains an integral component of comprehensive care. Adequate caloric intake, prevention of malnutrition, management of treatment-related symptoms, and referral to qualified dietitians can all improve supportive care. However, for most malignant and clonal hematologic disorders, evidence that specific foods, dietary regimens, or over-the-counter supplements function as disease-modifying therapy remains limited.

Research into selected micronutrients and biologically active compounds has generated intriguing findings, primarily in preclinical work or in carefully supervised clinical contexts. These data do not justify extrapolation to unsupervised supplementation or to commercial health claims of anticancer efficacy. Distinguishing supportive nutrition from disease-modifying therapy is therefore central to medical literacy and should be made explicit during clinical encounters.

## Vulnerability, Exploitation, and the Digital Amplification of Dietary Narratives

Serious diagnoses often produce fear and a perceived loss of control. In this psychological context, modifying diet may offer a tangible sense of agency. This search for agency can intersect with commercial incentives and digitally amplified messaging in ways that increase vulnerability.

In several jurisdictions, including Japan, criminal investigations have addressed group-based sales practices promoting unapproved supplements through emotionally manipulative techniques, sometimes including claims of anticancer efficacy ^(2)^. Regulatory authorities internationally have also reported concerns about misleading health claims and illegally marketed products disseminated through digital platforms ^(3), (4)^. The World Health Organization has described this broader environment as an “infodemic,” in which excessive or misleading health information circulates rapidly during periods of public anxiety ^(5)^. Algorithm-driven personalization and commercial optimization strategies may intensify exposure to emotionally resonant content. By the time patients enter the clinic, the question “What should I eat to get better?” may already be shaped by a curated digital information environment.

## Professional Responsibility and Public Medical Literacy

**Figure 1.**
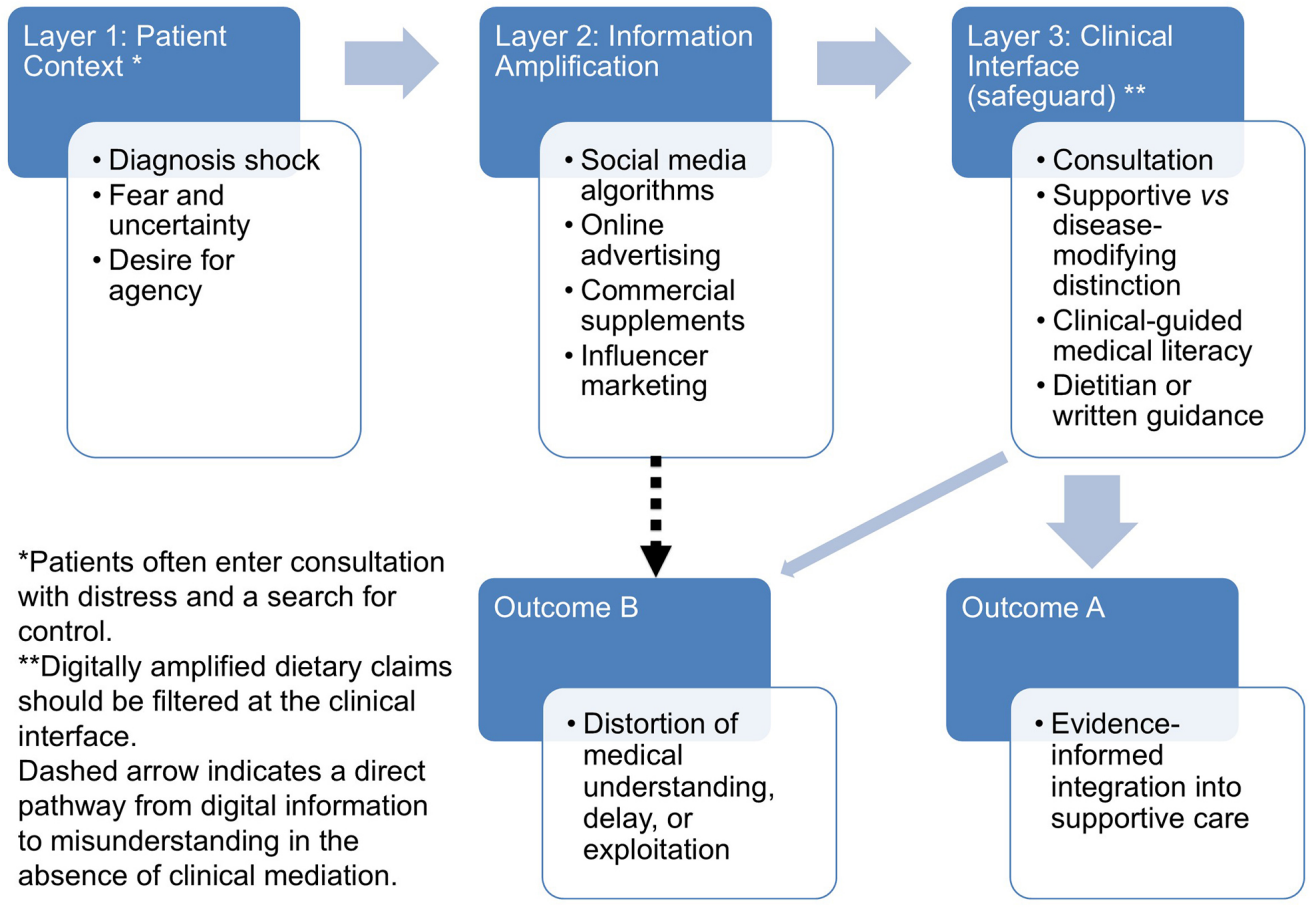
Digital amplification of dietary narratives and the clinician’s safeguard function. The figure illustrates three interacting layers: (1) patient context; (2) information amplification through digital and commercial channels; and (3) the clinical interface, where clinician-guided medical literacy functions as a safeguard or filter. This safeguard can redirect patients toward evidence-informed integration into supportive care (Outcome A), while interrupting the direct pathway from digitally amplified dietary narratives to distortion of medical understanding, delays in appropriate therapy, or exploitation (Outcome B). Patients newly diagnosed with hematologic diseases often seek dietary advice in the context of distress and uncertainty. Digitally amplified dietary claims may lead directly to misunderstanding or exploitation (dashed arrow) unless filtered at the clinical interface, where clinicians help distinguish supportive nutrition from disease-modifying therapy.

The clinical encounter represents a critical point of intervention within this ecosystem (Figure 1). Figure 1 conceptualizes the consultation not merely as a waypoint between information exposure and outcome, but as a safeguard layer. Layer 3 functions as a clinical filter: it can redirect patients toward evidence-informed supportive care while interrupting the pathway by which digitally amplified dietary narratives lead to misunderstanding, delays in appropriate therapy, financial exploitation, or both.

A practical response may be simple and direct. For example, when a patient with newly diagnosed acute leukemia asks whether a restrictive diet or a commercially promoted supplement can “fight the leukemia,” an effective answer may acknowledge the wish to contribute actively to recovery, explain that balanced nutrition can help maintain strength and tolerance to treatment, clarify that diet does not replace induction therapy, and advise against unverified products marketed with therapeutic claims. Offering dietitian support or written guidance can then preserve the sense of agency without reinforcing misinformation.

Practical steps may include providing clear explanations that distinguish supportive nutrition from disease-modifying therapy, offering explicit guidance regarding dietary supplements and commercially marketed health products, developing structured communication tools for use at diagnosis, and engaging proactively with media and patient advocacy organizations to counter misinformation. Strengthening medical literacy is therefore a concrete safeguard of patient safety in the digital era.

### Conclusions

When patients ask, “What should I eat to get better?” they express hope, fear, and a desire to participate in their own care. Respecting that question requires empathy and protecting patients requires clarity. In an era when health narratives circulate at unprecedented speed and scale, safeguarding patients demands not only scientific progress, but also deliberate public engagement and sustained stewardship of medical literacy.

## Article Information

### Acknowledgments

We are grateful to Ms. Mayumi Wada, Representative of the patient advocacy group *Moenokai*, for her thoughtful comments from a patient-centered perspective.

### Author Contributions

Akiyoshi Takami conceived the idea, drafted the manuscript, and approved the final version. Ichiro Hanamura critically revised the manuscript for important intellectual content and approved the final version. Both authors agree to be accountable for all aspects of the work.

### Conflicts of Interest

Akiyoshi Takami has received research funding from AIR WATER, lecture fees from Novartis Pharmaceuticals, and donation funding from Chugai Pharmaceutical and Kyowa Kirin. All relationships are unrelated to the content of this manuscript.

### Ethics Approval

Not applicable. This Opinion is based on professional experience and previously published literature, and did not involve new studies of human participants or identifiable patient data.

### Data Availability

Not applicable. No new datasets were generated or analyzed for this Perspective.
